# Association between body fat indices and female infertility: A cross-sectional study

**DOI:** 10.1097/MD.0000000000049327

**Published:** 2026-06-26

**Authors:** Li Huang, Wei Jing Yang, Gui Rong Kuang, Xiang Yan Li

**Affiliations:** a Academy for Educational Development and Innovation, The Education University of Hong Kong, Hong Kong SAR, People’s Republic of China; b Puding No. 2 Middle School, Anshun, Guizhou, People’s Republic of China; c Department of Gynecology, Puding County People’s Hospital, Anshun, Guizhou, People’s Republic of China; d Department of Reproductive Medicine, Anshun People’s Hospital, Anshun, Guizhou, People’s Republic of China.

**Keywords:** Conicity Index (C-index), infertility, Metabolic Score for Visceral Fat (METS-VF), NHANES, Relative Fat Mass Index (RFM)

## Abstract

Excess body fat and its distribution are known to influence female reproductive function; however, the relationship between emerging body fat-related indices and infertility remains insufficiently explored. This study aimed to investigate the association between the Conicity Index (C-index), Relative Fat Mass (RFM), and Metabolic Score for Visceral Fat (METS-VF) and infertility among women of reproductive age in the United States. The study included women aged 18 to 45 years and assessed infertility using the National Health and Nutrition Examination Survey question RHQ074 on reproductive health. Multivariable logistic regression, adjusted for confounders, was used to analyze the association between body fat indices and infertility risk. Receiver operating characteristic curve analysis was conducted to evaluate each metric’s predictive strength. Of 1103 women, 132 reported infertility. Infertile women had higher C-index (1.3 vs 1.27), RFM (43 vs 40), and METS-VF (8.24 vs 7.37) than fertile women. Each 1-unit increase in these indices was associated with higher odds of infertility (C-index odds ratio [OR] = 2.73, RFM OR = 1.07, METS-VF OR = 1.28). METS-VF had the highest predictive value (area under the curve = 0.65), as confirmed by random forest analysis. Positive associations between infertility and C-index, RFM, and METS-VF suggest that these indices could help assess infertility risk and support targeted reproductive health interventions.

## 1. Introduction

Infertility is the inability to achieve a successful pregnancy within 12 months among patients having regular, unprotected intercourse.^[1,2]^ It is a major global public health concern, affecting approximately 15% of couples globally, and its prevalence is increasing annually.^[3–5]^ Beyond reproductive challenges, infertility can strain relationships, leading to heightened anxiety, tension, and marital discord, sometimes even triggering marital crises. The financial burden of infertility treatments further exacerbates stress within affected families. Moreover, infertility is linked to an increased likelihood of reproductive malignancies and metabolic disorders.^[6–9]^

Obesity is a recognized contributing factor to infertility,^[10–12]^ and it is also intimately related to conditions such as diabetes and cardiovascular disease. Globally, obesity affects over 2 billion individuals, with a rising prevalence of obesity-related infertility among women. This condition, in which excess adipose tissue disrupts reproductive function through insulin resistance, hyperandrogenemia, and chronic inflammation, clinically manifests as anovulation, impaired endometrial receptivity, and reduced success rates in assisted reproductive technologies.^[13–15]^ Traditionally, a body mass index (BMI) >30 kg/m^2^ is considered indicative of obesity;^[16]^ however, BMI has limitations. While it can provide a general indication of body weight, it fails to discriminate between fat and muscle mass or assess fat distribution throughout the body. As a result, BMI alone is insufficient to accurately evaluate obesity and overall health, necessitating the consideration of multiple indicators for a more comprehensive assessment.^[3,17,18]^

Recent studies have explored more precise obesity metrics, such as the Conicity Index (C-index), Relative Fat Mass (RFM), and Metabolic Score for Visceral Fat (METS-VF), which have demonstrated strong predictive associations with type 2 diabetes mellitus.^[19–22]^ These indices incorporate anthropometric and metabolic parameters to provide more detailed information about body fat amount and distribution. The RFM provides a more accurate measure of total body fat than BMI, whereas the C-index evaluates fat distribution, especially the buildup of fat around the abdomen. The C-index is derived from waist circumference (WC), weight, and height and reflects central adiposity. METS-VF evaluates visceral fat accumulation, a key factor in central obesity, and is calculated based on metabolic indicators combined with anthropometric measurements.^[20,23,24]^

Despite these advancements, few studies have examined the association between female infertility and these emerging body fat-related indices. Thus, the present study aimed to investigate the association between C-index, RFM, and METS-VF and infertility among women of reproductive age using data from the National Health and Nutrition Examination Survey (NHANES) 2013 to 2018. We hypothesized that higher levels of these body fat-related indices would be associated with increased odds of infertility in women of reproductive age.

## 2. Methods

### 2.1. Data source

This cross-sectional analysis utilized information from NHANES, which is administered every 2 years by the Centers for Disease Control and Prevention’s National Center for Health Statistics. To assess the state of well-being and nutrition among Americans, NHANES uses a complex multi-tiered probability-based sampling technique.^[25]^ Data are amassed via in-home interrogations and physical checkups in mobile examination centers, covering demographic, dietary, laboratory, and examination information. The National Center for Health Statistics Ethics and Research Assessment Board approved the study’s methods, and participants’ informed consent was obtained. The public can obtain NHANES data at www.cdc.gov/nchs/nhanes/. This research adheres to Strengthening the Reporting of Observational Studies in Epidemiology reporting guidelines.^[26]^

### 2.2. Participant demographics

**Figure 1.**
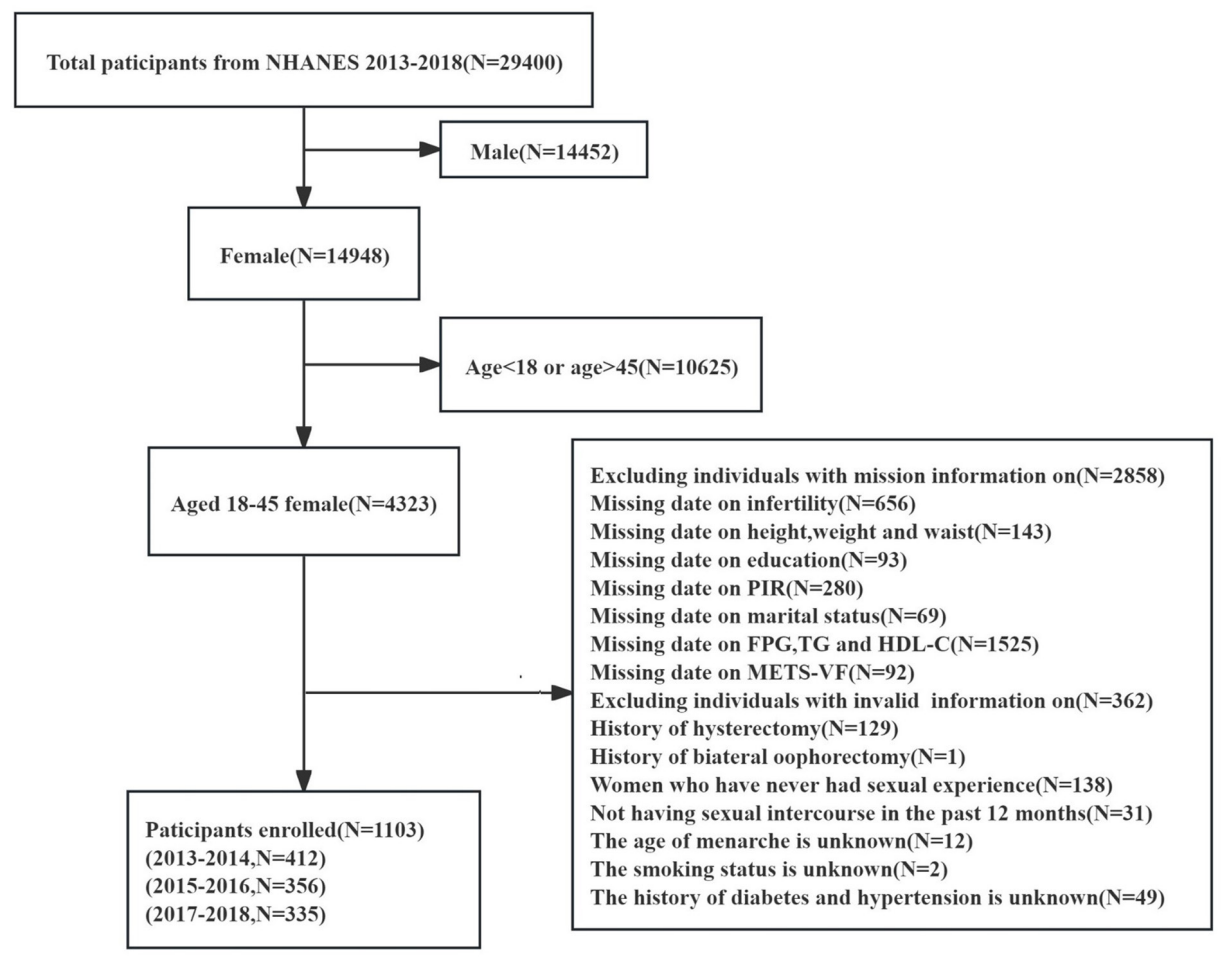
Flowchart for participants selection. FPG = fasting plasma glucose, HDL-C = high-density lipoprotein cholesterol, METS-VF = Metabolic Score for Visceral Fat, NHANES = National Health and Nutrition Examination Survey, PIR = poverty-to-income ratio, TG = triglycerides.

Data were obtained across 3 NHANES cycles: 2013 to 2014, 2015 to 2016, and 2017 to 2018, encompassing 29,400 participants. After excluding males, those aged younger than 18 years or older than 45 years, and those lacking data (e.g., height, weight, WC, or METS-VF), 1103 participants were incorporated in the ultimate assessment (Fig. 1).

### 2.3. Calculation of C-index, RFM, and METS-VF

Examination data were obtained from the Mobile Examination Center and recorded by trained health technicians. Height, WC, weight, high-density lipoprotein cholesterol, fasting plasma glucose, and triglycerides were determined following standardized protocols. The following formulas were used to calculate the body fat-related indices^[27–29]^:


$$C-index:\;0.109^{-1}\;WC\left(m\right){\left[\frac{Weight\left(kg\right)}{height\left(m\right)}\right]}^{-1/2},$$

$$\begin{array}{c} RFM:\;\left[70-20\times \left(\frac{height\left(m\right)}{waist\left(m\right)}\right)\right], \end{array}$$

$$\begin{array}{cc} & METS-VF:4.466+0.01\times {\left[\ln\left(\frac{\ln\left(2\times FPG+TG\right)\times BMI}{\ln\left(HDL-C\right)}\right)\right]}^{3}\\ & +3.329\times {\left[\ln\left(\frac{WC}{Height}\right)\right]}^{3}+0.319\times gender+0.594\times \ln\left(age\right). \end{array}$$

Participants were divided into tertiles (T1–T3) based on C-index, RFM, and METS-VF. Due to the small value of the C-index, when the C-index is regarded as a continuous variable, an exponential transformation was applied for analysis.

### 2.4. Assessment regarding infertility

The definition of infertility depended on responses to the question “Consider it you previously attempted for a year or more to conceive yet been unsuccessful?” A “Yes” response indicated infertility, while “No” indicated the absence of infertility.^[30]^

### 2.5. Covariates

Covariates included potential confounding factors such as age, race, and education level, age at menarche, regularity of menstruation, history of diabetes, history of hypertension, poverty-to-income ratio (PIR), total cholesterol, marital status, use of contraceptives, and smoking status. Weight was divided by height squared to calculate BMI. There were 3 classifications for the smoking status: the never smokers, who had smoked fewer than 100 cigarettes in their lives; the former smokers, who had smoked >100 cigarettes but were not smoking at the time; and the current smokers, who utilized cigarettes daily or sometimes.^[31]^ The educational levels were categorized in the following manner: those falling below the 9th Grade were identified as one group; those with a high school education or its equivalent were another group; and those who had completed college or more advanced studies were considered as the third group. The marital status was categorized in the following way: married people and those living with a spouse were categorized as being with a partner, whereas widowed, divorcing, separated, or had not been in a marriage before were classified as being without a partner.

### 2.6. Statistical analysis

To better represent the US population, every calculation was conducted using NHANES sample weighting. Categorical variables were described using unweighted sample sizes and weighted percentages, with the chi-square test used for comparisons. The reporting of continuous variables was done as mean ± standard deviation, with survey-weighted linear regression used for the purpose of comparing groups. The association between the C-index, RFM, and METS-VF and the risk of infertility was examined using a logistic regression model, adjusted for potential confounders: for Model I, age and race were the factors for adjustment. For Model II, the factors for adjustment included age, race, levels of education, marital status, cigarettes smoked, and the household PIR, age at menarche, regularity of menstruation, hypertensive history, and diabetic history; Model III further adjusted for fasting plasma glucose, triglycerides, total cholesterol, and history of using contraceptives. The nonlinear interactions were examined using restricted cubic splines with at least 3 knots among the 3 body fat-related indices and infertility. The knot values varied for each index, with specific knots located at (1.15, 1.26, 1.39) for C-index, (32.03, 40.48, 49.08) for RFM, and (5.35, 6.75, 7.81, 10.33) for METS-VF, and machine learning (the random forest algorithm) was used to rank the relative importance of each index. Receiver operating characteristic curves served the purpose of comparing the forecasting effectiveness across the indices.

All analyses were conducted using R (version 4.1.3; R Foundation for Statistical Computing). A threshold for statistical significance was established at *P* < .05.^[32]^

## 3. Results

### 3.1. Baseline characteristics

Among 1103 participants (unweighted), 132 females (12%) were identified as having infertility. The infertile females had a significantly higher mean age (35 years) compared with the fertile females (31 years; *P* < .001). Body fat-related indices showed significant differences between infertility and fertile females. The C-index was higher in the infertility females (1.3 vs 1.27, *P* < .001), as was the RFM (43 vs 40, *P* < .001) and METS-VF (8.24 vs 7.37, *P* < .001). Additionally, the infertile females had a higher WC (1.03 vs 0.94, *P* < .001), a higher waist-to-height ratio (0.63 vs 0.58, *P* < .001), and elevated prevalence rates of hypertension (21% vs 10%, *P* = .01) and diabetes (7.8% vs 2.4%, *P* = .01; Table 1).

**Table 1:** Characteristics of selected participants from the NHANES 2013 to 2018.

Characteristics	Fertile N = 971	Infertile N = 132	*P* value
Age (yr)	31 ± 8	35 ± 7	<.001
Race (%)			.2
Mexican American	153 (11)	20 (9.6)	
Other Hispanic	98 (7.9)	10 (5.0)	
Non-Hispanic White	337 (57)	49 (61)	
Non-Hispanic Black	217 (14)	30 (14)	
Non-Hispanic Asian	120 (6.2)	13 (3.5)	
Other race	46 (4.3)	10 (7.5)	
Education levels (%)			.10
<9th grade	152 (11)	21 (14)	
High school or equivalent	208 (21)	23 (14)	
College or over	611 (68)	88 (72)	
Marital status (%)			<.001
With partner	604 (66)	99 (80)	
Without partner	367 (34)	33 (20)	
PIR	2.61 ± 1.65	2.88 ± 1.70	.13
Smoking status (%)			.3
Never	703 (69)	92 (66)	
Former	107 (13)	22 (20)	
Current	161 (18)	18 (14)	
BMI (%)			<.001
≤25 kg/m^2^	405 (42)	35 (26)	
25–30 kg/m^2^	232 (24)	20 (15)	
>30 kg/m^2^	334 (35)	77 (60)	
WC (m)	0.94 ± 0.19	1.03 ± 0.19	<.001
WHtR	0.58 ± 0.11	0.63 ± 0.11	<.001
HDL-C (mmol/L)	1.57 ± 0.40	1.44 ± 0.33	.01
TG (mmol/L)	1.11 ± 2.03	1.06 ± 0.48	.2
FBG (mmol/L)	5.38 ± 1.03	5.60 ± 1.71	.6
Total cholesterol (mg/dL)	182 ± 39	180 ± 34	.7
C-index	1.27 ± 0.09	1.30 ± 0.09	<.001
RFM	40 ± 6	43 ± 6	<.001
METS-VF	7.37 ± 1.58	8.24 ± 1.91	<.001
Age at menarche (yr)	12.63 ± 1.69	12.20 ± 1.73	.04
Menstrual regularity (%)			.02
Yes	905 (93)	121 (85)	
No	66 (6.7)	11 (15)	
Contraceptive drug use			>.9
Yes	666 (76)	93 (76)	
No	305 (24)	39 (24)	
Hypertension (%)			.01
Yes	108 (10)	30 (21)	
No	863 (90)	102 (79)	
Diabetes (%)			.01
Yes	29 (2.4)	11 (7.8)	
No	942 (98)	121 (92)	

The weighted mean ± standard error for continuous variables and unweighted counts (weighted percentage) for categorical variables were calculated. The *P* value was obtained through survey-weighted linear regression or weighted Chi-square test.

BMI = body mass index, FBG = fasting blood glucose, HDL-C = high-density lipoprotein cholesterol, METS-VF = Metabolic Score for Visceral Fat, NHANES = National Health and Nutrition Examination Survey, PIR = poverty-to-income ratio, RFM = Relative Fat Mass, WC = waist circumference, WHtR = waist-to-height ratio.

### 3.2. Association between body fat-related indices and infertility

**Figure 2.**
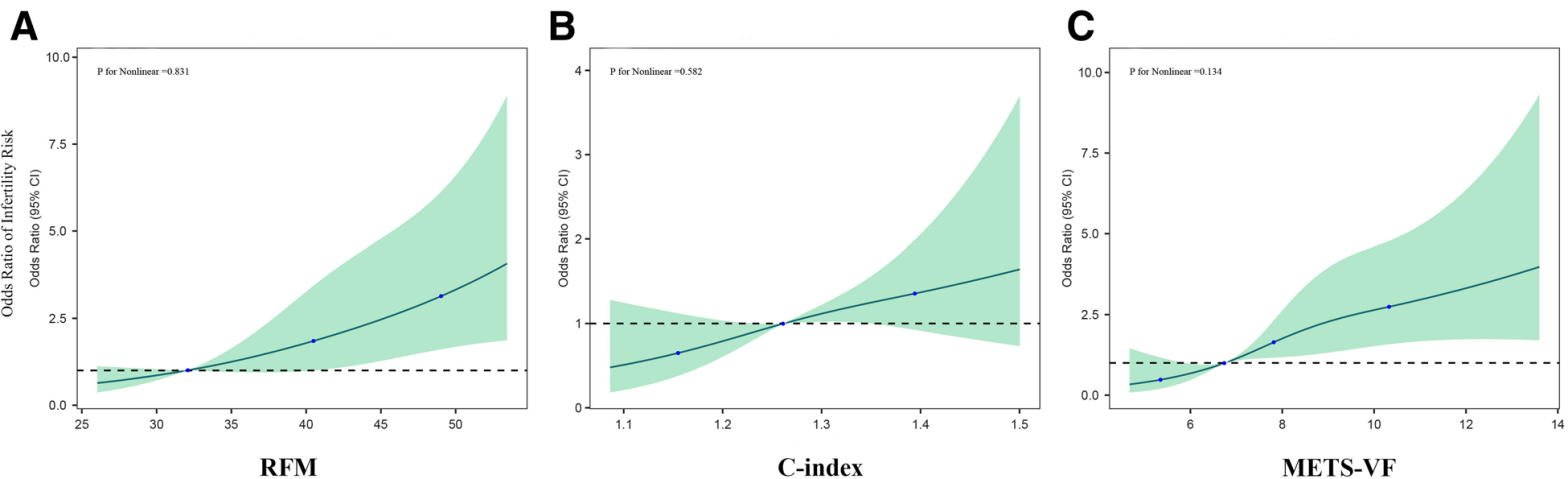
The weighted nonlinear association between RFM, C-index, and METS-VF and female infertility was analyzed using a weighted restricted cubic spline model. ORs (blue line) and 95% CIs (green shade area) were calculated after adjustment for age, race, educational levels, marital status, smoking status, history of hypertension, history of diabetes, PIR, age at menarche, menstrual regularity, total cholesterol, fasting blood glucose levels, triglyceride levels, and contraceptive drug use. The dashed line indicates the reference line of OR = 1. Age, triglyceride, and fasting blood glucose were not adjusted in the evaluation of the correlation between METS-VF and infertility. (A) Weighted RCS curve for RFM and infertility (B) Weighted RCS curve for C-index and infertility (C) Weighted RCS curve for METS-VF and infertility. C-index = Conicity Index, CI = confidence interval, METS-VF = Metabolic Score for Visceral Fat, OR = odds ratio, PIR = poverty-to-income ratio, RFM = Relative Fat Mass.

Both univariate and multivariate analyses showed that all 3 body fat-related indices (C-index, RFM, and METS-VF) were positively associated with female infertility. For every 1-unit increment in C-index, the incidence of infertility grew by 2.10-fold (odds ratio [OR] = 2.10, 95% confidence interval [CI]: 1.01–4.37; *P* = .048; Table 2). Infertility risk grew by 7% (OR = 1.07, 95% CI: 1.02–1.10; *P* = .004) for every unit increase in RFM. A 1-unit addition in METS-VF increased risk of infertility by 28% (OR = 1.28, 95% CI: 1.12–1.46; *P* < .001). According to categorical analysis based on tertiles, the risk of infertility was 1.88 times higher for the highest tertile of the C-index than for the lowest tertile (OR = 1.88, 95% CI: 1.02–3.08; *P* = .04). Likewise, the highest tertile of RFM was related to a 2.53 times greater infertility risk (OR = 2.53, 95% CI: 1.26–5.07; *P* = .01). With respect to METS-VF, the infertility risk in the highest tertile was 3.05 times greater (OR = 3.05, 95% CI: 1.74–5.34; *P* < .001). *P* trends for all these indices were statistically significant (all *P* < .05). Restricted cubic splines further confirmed a linear relationship, like a J-shaped connection, between C-index, RFM, METS-VF, and infertility (*P* for nonlinear > 0.05; Fig. 2).

**Table 2:** Association between body fat-related indices and female infertility.

Body fat-related indices	Crude model		Model I		Model II		Model III	
	OR (95% CI)	*P* value	OR (95% CI)	*P* value	OR (95% CI)	*P* value	OR (95% CI)	*P* value
C-index	2.72 (1.52–4.86)	<.001	2.12 (1.11–4.06)	.02	1.73 (0.89–3.36)	.10	2.10 (1.01–4.37)	.048
T1	Ref.		Ref.		Ref.		Ref.	
T2	1.80 (1.10–2.95)	.02	1.47 (0.90–2.39)	.12	1.39 (0.82–2.34)	.21	1.49 (0.88–2.50)	.13
T3	2.43 (1.43–4.11)	.002	1.86 (1.07–3.24)	.03	1.62 (0.90–2.91)	.10	1.88 (1.02–3.48)	.04
*P* for trend		.002		.03		.11		.047
RFM	1.08 (1.04–1.11)	<.001	1.06 (1.02–1.10)	.004	1.06 (1.02–1.10)	.006	1.07 (1.02–1.10)	.004
T1	Ref.		Ref.		Ref.		Ref.	
T2	1.35 (0.72–2.53)	.34	1.08 (0.56–2.08)	.82	1.10 (0.60–2.04)	.75	1.21 (0.66–2.23)	.52
T3	2.77 (1.55–4.96)	.001	2.16 (1.12–4.16)	.02	2.12 (1.14–3.97)	.02	2.53 (1.26–5.07)	.01
*P* for trend		.001		.02		.02		.01
METS-VF*	1.3 (1.15–1.48)	<.001	1.30 (1.15–1.48)	<.001	1.28 (1.12–1.46)	<.001	1.28 (1.12–1.46)	<.001
T1	Ref.		Ref.		Ref.		Ref.	
T2	2.03 (1.07–3.84)	.03	2.07 (1.09–3.92)	.03	1.98 (1.07–3.65)	.03	2.03 (1.10–3.76)	.03
T3	3.23 (1.86–5.60)	<.001	3.22 (1.82–5.70)	<.001	3.05 (1.73–5.36)	<.001	3.05 (1.74–5.34)	<.001
*P* for trend		<.001		<.001		<.001		<.001

Crude model: unadjusted model.

Model I: adjusted for age and race.

Model II: adjusted for age, race, PIR, educational level, marital status, smoking status, hypertension, diabetes, age at menarche, and menstrual regularity.

Model III: further adjusted for TC, TG, FBG, and contraceptive drug use.

For the C-index, group T1 is 1.0297 ≤ C-index ≤ 1.2042, group T2 is 1.2214 ≤ C-index ≤ 1.3051, and group T3 is 1.3052 ≤ C-index ≤ 1.5914. For RFM, group T1 is 22.6772 ≤ RFM ≤ 37.4503, group T2 is 37.4730 ≤ RFM ≤ 43.8253, and group T3 is 43.8261 ≤ RFM ≤ 58.4116. For METS-VF, group T1 is 4.0735 ≤ METS-VF ≤ 6.7025, group T2 is 6.7053 ≤ METS-VF ≤ 7.8799, and group T3 is 7.9106 ≤ METS-VF ≤ 15.4987.

C-index = Conicity Index, CI = confidence interval, FBG = fasting blood glucose, METS-VF = Metabolic Score for Visceral Fat, OR = odds ratio, PIR = poverty-to-income ratio, Ref. = reference, RFM = Relative Fat Mass, TC = total cholesterol, TG = triglycerides.

* Age, TG, and FBG were not adjusted in all models.

### 3.3. Subgroup analyses

**Figure 3.**
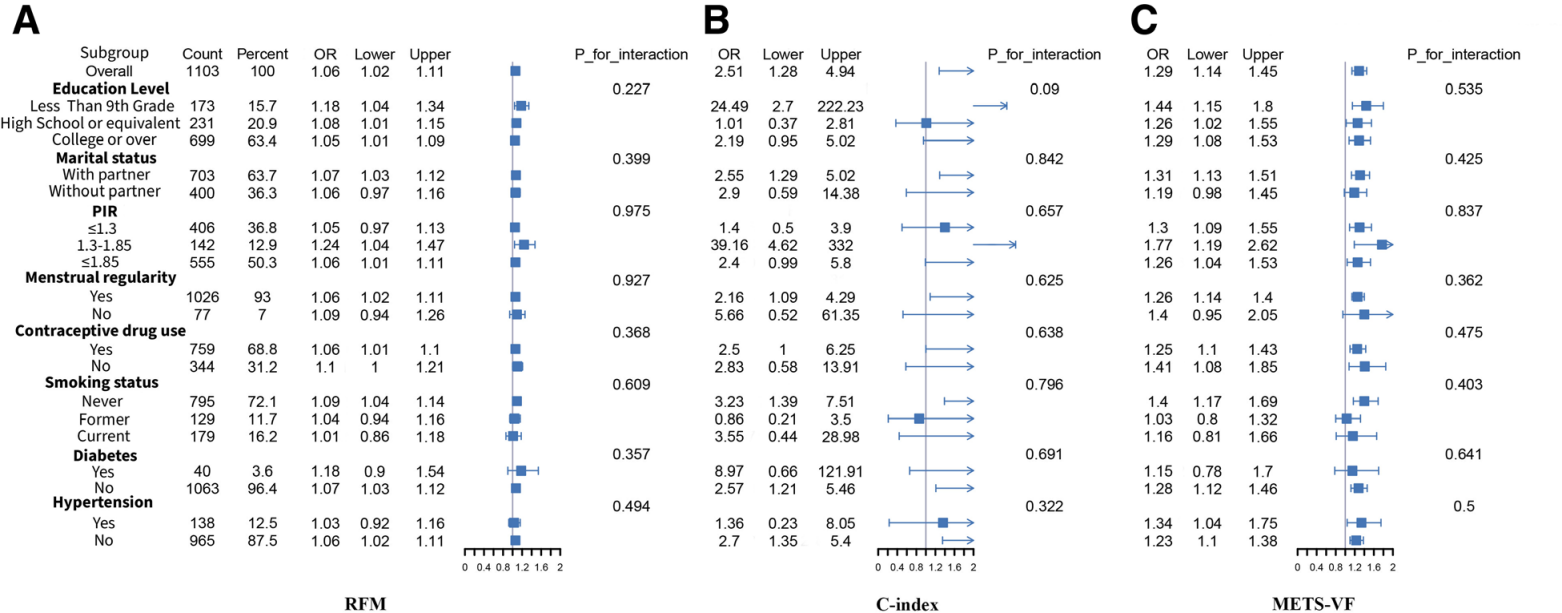
Weighted subgroup analyses were conducted to examine the associations of body fat-related indices with female infertility. (A) RFM; (B) C-index; and (C) METS-VF. All odds ratios (ORs) represent adjusted estimates, with the reference group defined as fertile individuals within each subgroup. Panel A was adjusted for age, TC, TG, fasting blood glucose (FBG), and age at menarche; panel B included the same covariates as panel A; panel C was adjusted for TC and age at menarche only. C-index = Conicity Index, METS-VF = Metabolic Score for Visceral Fat, RFM = Relative Fat Mass, TC = total cholesterol, TG = triglycerides.

Stratified by education levels, smoking status, marital status, hypertensive history, diabetic history, PIR, menstrual regularity, and contraceptive use, subgroup analyses revealed no statistically significant interactions between C-index, RFM, METS-VF, and female reproductive failure across different subgroups (Fig. 3).

### 3.4. Results from the random forest model algorithm

**Figure 4.**
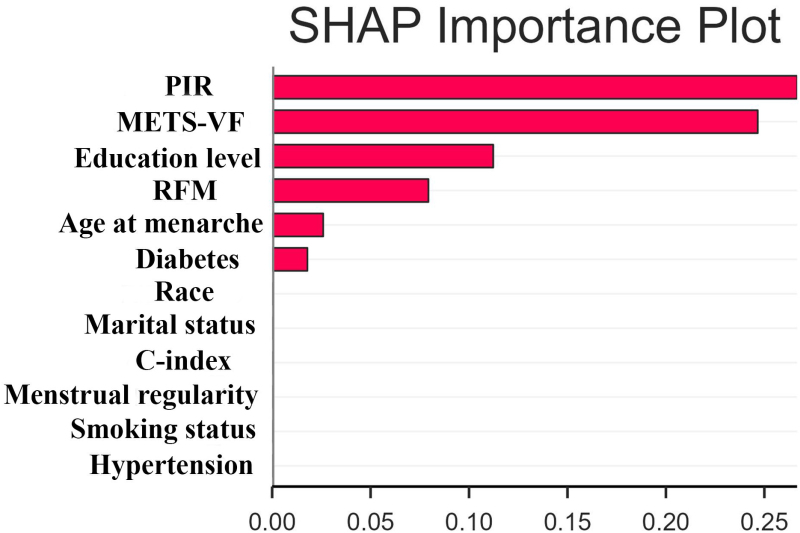
Results of the random forest algorithm. Random forest is a decision tree-based ensemble method. C-index = Conicity Index, METS-VF = Metabolic Score for Visceral Fat, PIR = poverty-to-income ratio, RFM = Relative Fat Mass, SHAP = shapley additive explanations.

Using the decision tree-based machine learning method, random forest, METS-VF was found to be the most significant predictor of infertility risk, followed by RFM and C-index (Fig. 4).

### 3.5. Receiver operating characteristic curve-based analytics

**Figure 5.**
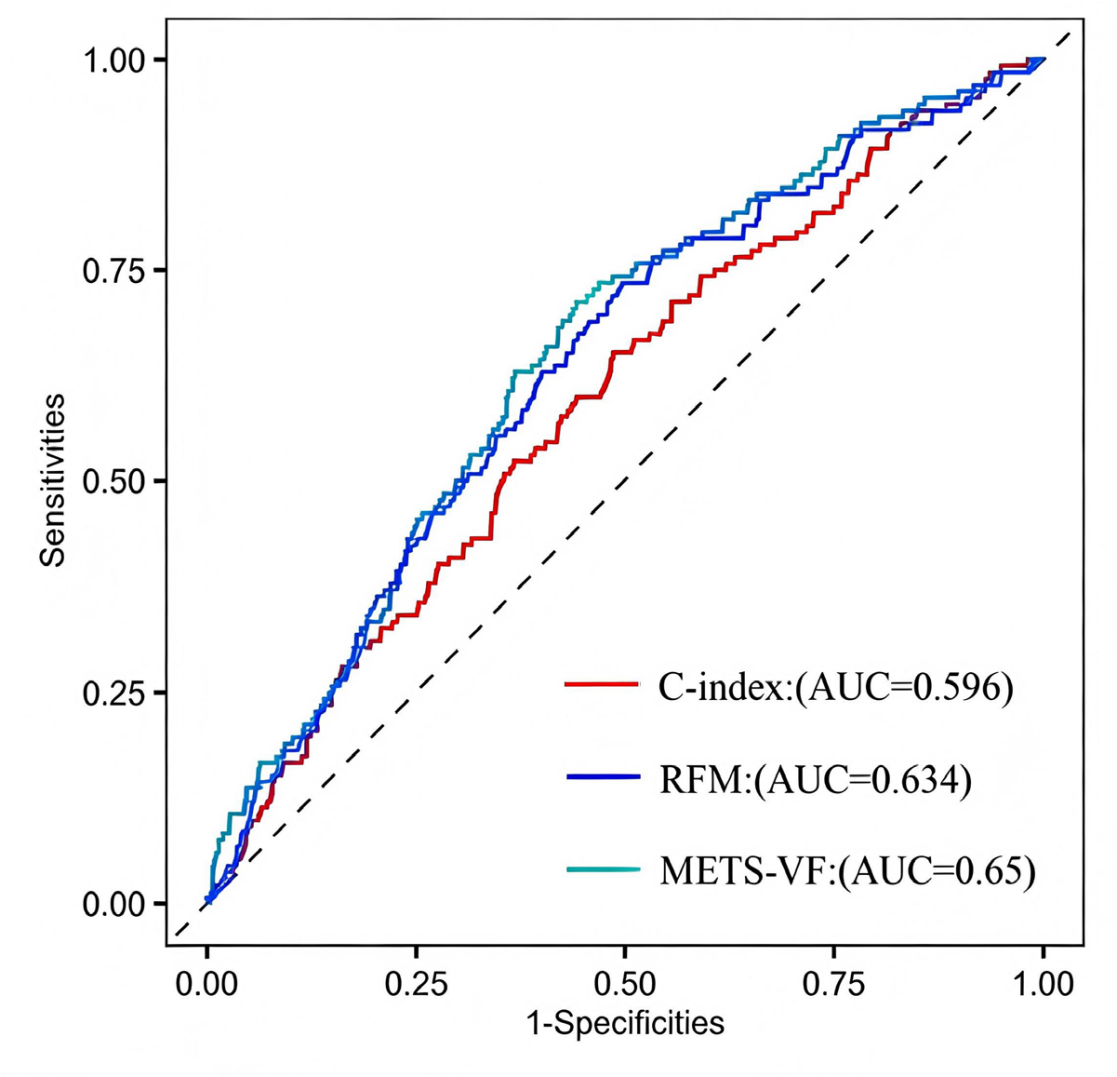
ROC curves of 3 body fat-related indices for predicting female infertility. AUC = area under the curve, C-index = Conicity Index, METS-VF = Metabolic Score for Visceral Fat, RFM = Relative Fat Mass, ROC = receiver operating characteristic.

METS-VF was shown to have the highest predictive statistic for infertility based on the area under the curve (AUC = 0.65, 95% CI: 0.6011–0.6980), followed by RFM (AUC = 0.634, 95% CI: 0.5846–0.6835), and C-index (AUC = 0.596, 95% CI: 0.5451–0.6468). There were no statistically significant differences between METS-VF and RFM (*P* = .24; Fig. 5).

## 4. Discussion

This study, based on data from 3 cycles (2013–2018), provides compelling evidence that increased values of C-index, RFM, and METS-VF are considerably associated with higher infertility risk in women. Among them, METS-VF was identified as the most significant predictor of infertility risk, followed by RFM and C-index. These results suggest that managing these body fat-related indices could help mitigate infertility risk.

Previous studies have also highlighted the impact of obesity on infertility, as well as its association with long-term conditions such as diabetes, hypertension, and cardiovascular disease, and reproductive health issues,^[33–35]^ including endometriosis, ovarian cancer, polycystic ovary syndrome, and irregular menstruation.^[36,37]^ Consistent with previous conclusions, higher levels of body fat contribute to a doubled infertility risk in obese women as opposed to their nonobese counterparts.^[38]^ Notably, weight loss interventions have improved reproductive outcomes by modulating hormonal imbalances and improving oocyte quality in obese women. Previous studies have illustrated that in overweight or obese women undergoing assisted reproductive and other treatments, the quality of oocytes and treatment outcomes were unsatisfactory to a certain extent. However, after losing weight, the outcome would improve.^[39]^ This could be due to dysfunction in the hypothalamic-pituitary-gonadal axis among obese women.^[40]^ This effect is likely due to dysregulation of the hypothalamic-pituitary-gonadal axis and restoration of hormonal balance.^[41]^

Although BMI is frequently used to evaluate obesity, it has limitations in distinguishing between fat and muscle mass and in detecting visceral fat.^[42]^ In contrast, the C-index, RFM, and METS-VF offer a more accurate understanding of body fat distribution and metabolic health.^[43,44]^ Specifically, the C-index reflects abdominal fat accumulation, RFM estimates body fat percentage, and METS-VF incorporates metabolic and age-related factors, making it a more comprehensive indicator of visceral fat and metabolic syndrome.^[45–47]^ Our findings highlight the utility of these indices, particularly METS-VF, as predictors of infertility.

The simplicity and cost-effectiveness of measuring these indices make them practical tools for assessing infertility risk. Considering the increasing prevalence of obesity globally and its influence on reproductive health, these indices could be integrated into routine clinical assessments to guide interventions aimed at reducing infertility risk through weight management, dietary improvements, and increased physical activity.

Certain limitations must be acknowledged. As this is a cross-sectional study, it is unable to determine causality between body fat indices and infertility. Moreover, the NHANES database lacks information on important factors such as partner infertility status and the duration of infertility, which could further influence the results. Finally, these results might not be applicable to populations outside the United States, necessitating further research in diverse cohorts.

## 5. Conclusion

Our study suggests a positive association between all 3 body fat-related indices (C-index, RFM, and METS-VF) and female infertility. These easily measurable indices may provide valuable insights for infertility risk assessment and could help inform interventions aimed at reducing body fat and improving metabolic health to enhance reproductive outcomes.

Further longitudinal and mechanistic studies are needed to clarify the causal relationships and to explore the underlying biological mechanisms linking body fat distribution and female infertility.

## Author contributions

**Conceptualization:** Li Huang, Wei Jing Yang.

**Data curation:** Li Huang, Wei Jing Yang.

**Formal analysis:** Li Huang, Wei Jing Yang.

**Investigation:** Li Huang, Wei Jing Yang.

**Methodology:** Li Huang, Wei Jing Yang.

**Project administration:** Li Huang, Wei Jing Yang.

**Resources:** Li Huang, Wei Jing Yang.

**Software:** Li Huang, Wei Jing Yang.

**Supervision:** Li Huang, Wei Jing Yang.

**Validation:** Li Huang, Wei Jing Yang.

**Visualization:** Li Huang, Wei Jing Yang.

**Writing – original draft:** Li Huang, Wei Jing Yang.

**Writing – review & editing:** Li Huang, Wei Jing Yang, Gui Rong Kuang, Xiang Yan Li.
